# Supplementary material for: Comprehensive Annotation and Functional Exploration of MicroRNAs in Lettuce
Source: Front Plant Sci. 2021 Dec 24;12:781836. doi: 10.3389/fpls.2021.781836 (PMC8739914; doi:10.3389/fpls.2021.781836)
Supplement: Supplementary file 2 [file Data_Sheet_8.ZIP › GSM2230748.plot/Lsa-miR397_Lsat_1_v5_gn_2_128840.1_467_TPlot.pdf]

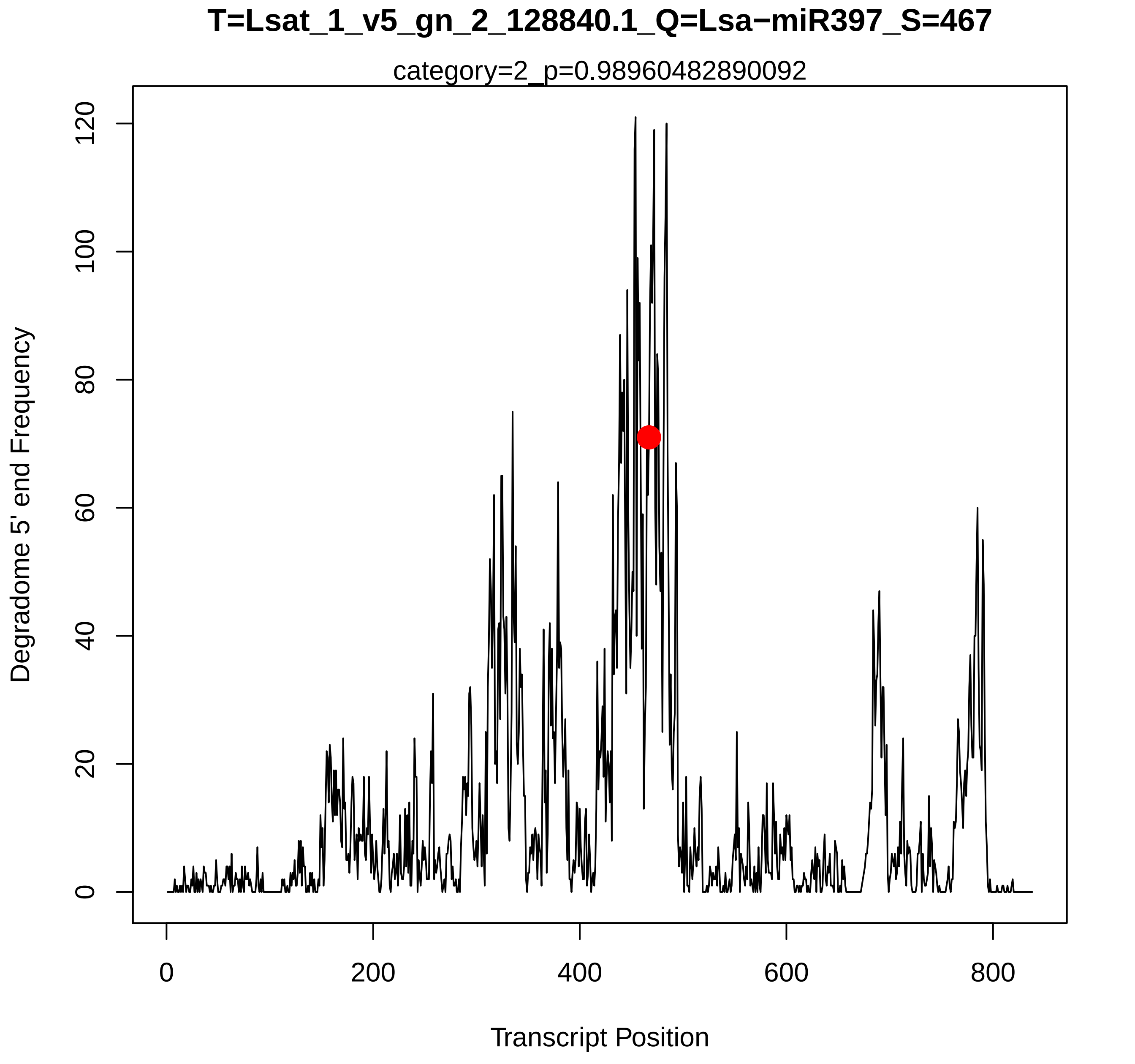

T=Lsat_1_v5_gn_2_128840.1_Q=Lsa−miR397_S=467
category=2_p=0.98960482890092
Degradome 5' end Frequency
Transcript Position
0
20
40
60
80
100
120
0
200
400
600
800
